# Black Edible Films from Protein-Containing Defatted Cake of *Nigella sativa* Seeds

**DOI:** 10.3390/ijms21030832

**Published:** 2020-01-28

**Authors:** Mohammed Sabbah, Mohammad Altamimi, Prospero Di Pierro, Chiara Schiraldi, Marcella Cammarota, Raffaele Porta

**Affiliations:** 1 Department of Nutrition and Food Technology, An-Najah National University, Nablus P.O. Box: 7, Palestine; m.sabbah@najah.edu (M.S.); m.altamimi@najah.edu (M.A.); 2 Department of Chemical Sciences, University of Naples “Federico II”, 80126 Naples, Italy; dipierro@unina.it; 3 Department of Experimental Medicine, Section of Biotechnology and Molecular Biology, University of Campania “Luigi Vanvitelli”, 80138 Naples, Italy; chiara.schiraldi@unicampania.it (C.S.); marcella.cammarota@unicampania.it (M.C.)

**Keywords:** *Nigella sativa*, protein-based films, industrial biorenewables, oil-seed waste, transglutaminase

## Abstract

Black biodegradable/edible protein-based films were prepared from defatted cake waste obtained from *Nigella sativa* (black cumin) seeds as by-product of oil extraction process. The effects of pH, glycerol concentrations, and transglutaminase-catalyzed protein cross-linking activity on the stability of film-forming solutions were studied to determine the best experimental conditions to produce handleable films. Proteins contained in the analyzed defatted cake were shown to be able to act as transglutaminase acyl donor and acceptor substrates being polymerized when incubated in vitro in the presence of the enzyme. Film-forming solutions containing 20% glycerol and casted at pH 8.0 after treatment with the enzyme gave rise to morphologically more homogeneous films possessing mechanical and barrier properties, as well as antimicrobial activity, compatible with their possible applications as food packaging materials and mulching sheets. These findings confirm the validity of the strategy to consider the seed oil processed cakes as protein-based renewable sources to produce not only fertilizers, animal feed, or culinary food but also further valuable products such as bioplastics.

## 1. Introduction

It is now generally recognized that the environmental pollution due to plastic polymers is a global concern. Landfills and oceans are the main destination of the plastic waste, whereas only less than 10% of plastics produced every year is recycled [1]. Seeking biodegradable alternatives for plastics is increasing, even though they still probably represent less than 5% of the total plastic materials used today. 

For the food industry, packaging is an important process to protect food items from the external environment and to extend their shelf life. Packaging materials are an effective barrier against light, heat, moisture, gases, and microorganisms, as well as they prevent loss of flavors and aromas. Oil-derived plastics, mostly thanks to their versatile characteristics of stability, flexibility, rigidity, transparency, and molding, have been and continue to be extensively used to protect the majority of the commercialized foods. Plastic packaging wastes represent more than 15% and 25% of total solid wastes in the USA and Europe, respectively [2]. Nevertheless, both the demand and the production for new biodegradable packaging materials able to substitute for the traditional plastic ones are on the rise [1].

The current focus of many researchers is on the use of bio-polymers obtained from agri-food by-products as potential alternatives to fossil-based polymers. These biomacromolecular sources are not only safe for humans but also environmentally friendly due to their biodegradability. Numerous food industry wastes, such as citrus peels, cassava by-products, and apple pomace, as well as whey, have been already proposed as possible biodegradable sources to produce alternative materials useful for food packaging [3,4,5,6,7]. 

Different seeds are widely used to produce vegetable oils or animal feed. Those from soy, sesame, and bitter vetch have been shown to be potential sources of biodegradable/edible films [8,9,10,11,12]. In fact, after oil extraction, the leftover cakes may contain up to 50% recyclable proteins that may produce bio-materials. Moreover, the defatted seed wastes generally contain a high level of polyphenols and, thus, they may even act as antioxidants and antimicrobials inside the derived edible films used as food coatings or wrappings. Consequently, the packaged food products are preserved from deterioration (rancidity, oxidation, and spoilage by microorganisms). This may increase the total polyphenols ingested by the consumer giving an additional nutritional contribution of these films [13,14]. 

*Nigella sativa* (black cumin), belonging to *Ranunculaceae* family, is cultivated in various parts of the world and, in particular, is widely grown in the Middle East countries [15]. It is a bushy, self-branching plant with either white or pale to dark blue flowers [16]. Its seeds are mainly used as condiment, while the extracted oil is exploited in traditional medicine for the treatment of a wide range of diseases. Seed composition varies from one geographical area to another, mainly because of the different environmental factors and horticultural practices. On average, the seeds contain 21% proteins, 35% fat, 29% carbohydrates, 6% crude fibers, 5% moisture, and 4% ash [15].

Although several researches have been performed to study the benefits of *Nigella sativa* seeds, roots, and shoots, only few data are available on the bioactivity of its seed cake and, among these, the high antioxidant activity probably due to the predominant phenolic compounds (hydroxybenzoic, syringic, and *p*-coumaric acids) [17]. *Nigella sativa* seeds have been in use for 3000 years, but the precise global consumption of them, as well as of the extracted oil, is not known. It is estimated that India alone produces 85% of the world’s cumin (brown and black), with 250,000 metric tons/year, followed by Middle Eastern countries including Syria, Turkey, and Egypt. Major importers are Asian-Pacific countries, North America, EU, South Africa, and Middle Eastern countries. In addition to the seeds, also the extracted oil is exported worldwide [18].

With the increasing awareness about its therapeutic benefits, the demand on black cumin oil is rising. Few bioactive compounds extracted from *Nigella sativa* seeds have been identified, the most important being the thymoquinones [19]. Other phytochemicals from different varieties of *Nigella sativa* include sterols, saponins, phenolic compounds, various alkaloids, some lipid constituents, and fatty acids, as well as volatile oils of different composition [20]. Furthermore, the most important compounds responsible for seed pigmentation are phytomelanins, high-molecular weight polymers formed by the oxidation of phenols [21,22,23]. It has been highlighted that the black coated seeds survive for years under the soil and are resistant to soil acidity, insect predators, and microbial attack. 

Currently, to the authors’ knowledge, there is no report about the amount of black cumin seeds utilized for oil extraction. Large quantities of defatted cake is certainly generated as by-product and most of it is offered as animal feed due to the high protein content [24]. Moreover, there are no reports in the literature about the exploitation of the proteins extracted from *Nigella sativa* defatted seed cake (*Ns*DSC) to obtain novel bio-materials.

In this study, black films were prepared from a protein concentrate (PC) derived from *Ns*DSC, in the presence of glycerol (GLY) as plasticizer, and their characterization was carried out. Furthermore, the effect of transglutaminase (TGase), a food-grade protein cross-linking enzyme used as processing aid [25,26,27], was also investigated in the attempt to improve the functional properties of the obtained biodegradable/edible material.

## 2. Results and Discussion 

### 2.1. FFS Zeta Potential and Z-Average Measurements

**Figure 1:**
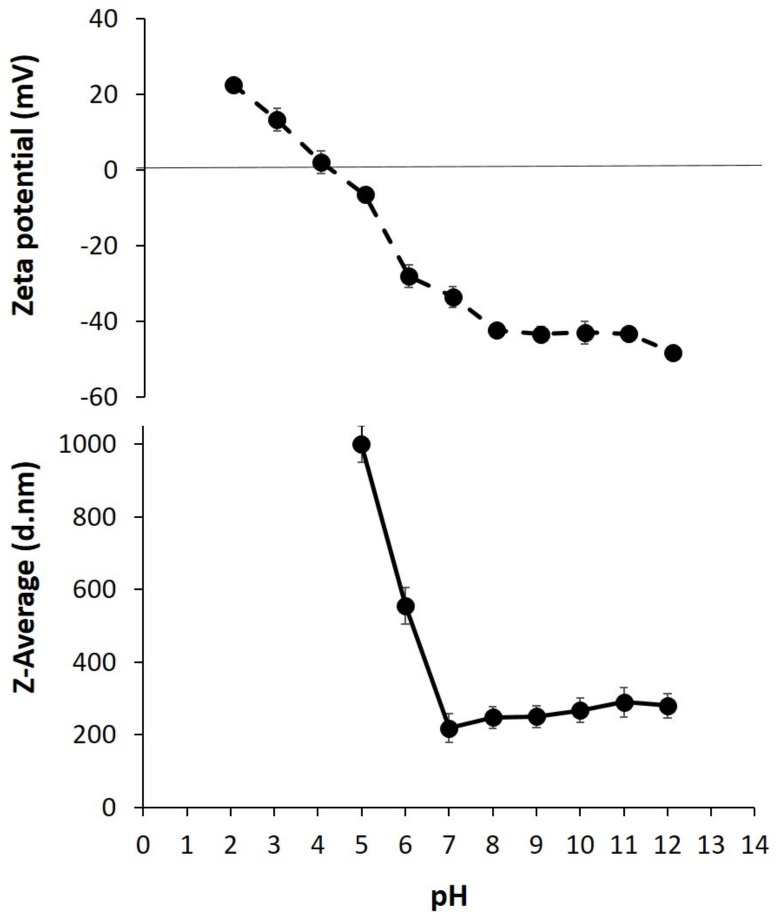
Zeta potential (**upper panel**) and Z-average size (**lower panel**) measurements of *Nigella sativa* defatted seed cake (*Ns*DSC)-derived protein concentrate (PC) performed at different pH values. PC sample contained 1.0 mg protein dissolved in 1.0 mL of distilled water at pH 12. All the results were reported as mean ± standard deviation. Further experimental details are given in the text.

Zeta potential, a quantitative parameter monitoring the micro- and nano-particle mobility in an electrical field, as well as the size of the particles occurring in aqueous solution of PC obtained from *Ns*DSC, were preliminarily determined as a function of pH to find proper conditions for the development of stable film-forming solutions (FFSs) [28]. The zeta potential of *Ns*DSC-derived proteins showed a value ranging from −45 mV to −41 mV between pH 12.0 and 8.0 (Figure 1, upper panel), whereas the value was found to progressively decrease to −27 mV and −5 mV with lower pH 6.0 and 5.0, respectively, then becoming positive under pH 4.0. Therefore, these findings clearly suggested to prepare protein FFSs at pH values no lower than 6.0 in order to obtain stable solutions. This indication was also confirmed by the Z-average size of the protein particles which lost their nano-dimension under pH 6.0 reaching at lower pHs a Z-average value over 1000 d.nm (diameter nanometers) (Figure 1, lower panel).

### 2.2. NsDSC Proteins as TGase Substrates

**Figure 2:**
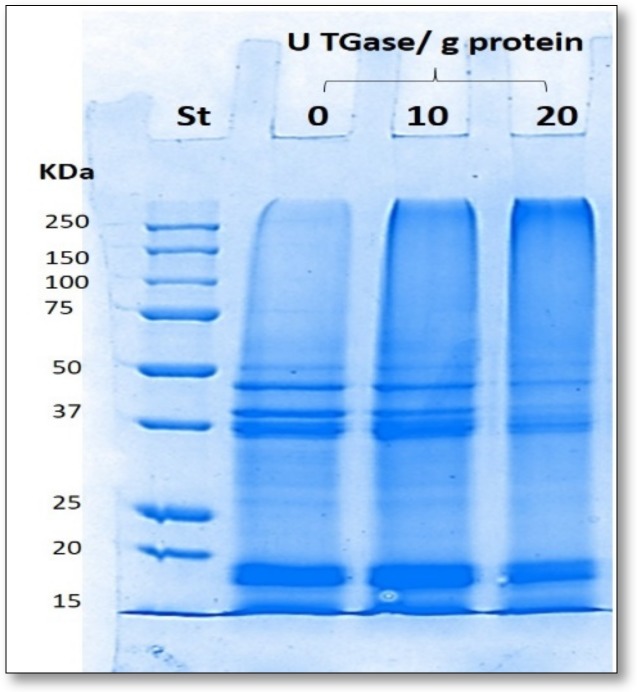
SDS-PAGE profile of film forming solutions (FFSs) previously incubated in the absence or presence of two different amounts of transglutaminase (TGase). Further experimental details are given in the text.

It was previously demonstrated that different seed proteins were able to act as TGase substrates [8,11,12,29,30]. Therefore, we verified whether one or more proteins occurring in PC obtained from *Ns*DSC were able to be cross-linked during incubation at 37 °C for 2 h at pH 8.0 in the presence of different amounts of the enzyme. Aliquots of reaction mixtures were then analyzed by SDS-PAGE and the extent of the enzymatic protein cross-linking was evaluated observing the progressive decrease of intensity of the Coomassie-stained bands occurring in the electrophoretic protein profiles. Reaction mixtures prepared in the absence of TGase were also incubated to confirm the enzyme-dependent polymer formation. As shown in Figure 2, a progressive attenuation of all proteins contained in the *Ns*DSC was observed when the reaction mixtures were incubated in the presence of increasing concentrations of TGase, while a concomitant formation of high mol. wt. polymers became detectable at the top of the gel. These results indicate that, similarly to other seed proteins [8,11,31], both glutamine and lysine reactive residues able to give rise to cross-linked homo- and/or hetero-polymers in the presence of the enzyme were present into the proteins contained in *Ns*DSC.

**Figure 3:**
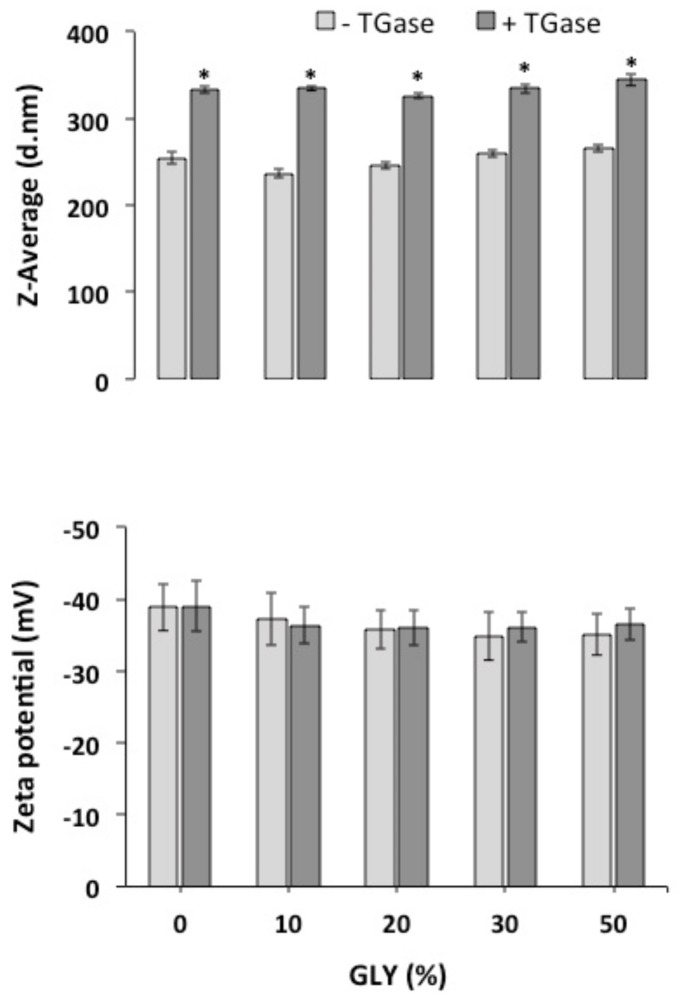
Z-Average and zeta potential, measured at pH 8.0, of protein concentrate (PC, 400 mg) FFSs containing different concentrations of glycerol (GLY) after incubation in the absence or presence of transglutaminase (TGase, 20 Units/g protein). Further experimental details are given in the text. * Significantly different values as compared to the ones obtained under the same experimental conditions in the absence of TGase (*p* < 0.05).

Finally, experiments aimed to find adequate conditions for the development of solutions able to give rise to plasticized films were carried out in the presence of different concentrations of GLY. Thus, both zeta potential and Z-average of FFSs, containing PC previously incubated in the absence or presence of TGase, were measured after GLY addition. The results reported in Figure 3 show that neither GLY nor enzymatic treatment significantly influenced FFS zeta potential, whereas the Z-average values slightly increased in the samples previously incubated with TGase, independently on the presence of GLY, probably as a consequence of protein cross-linking.

### 2.3. Black Edible Films from PC of NsDSC

**Figure 4:**
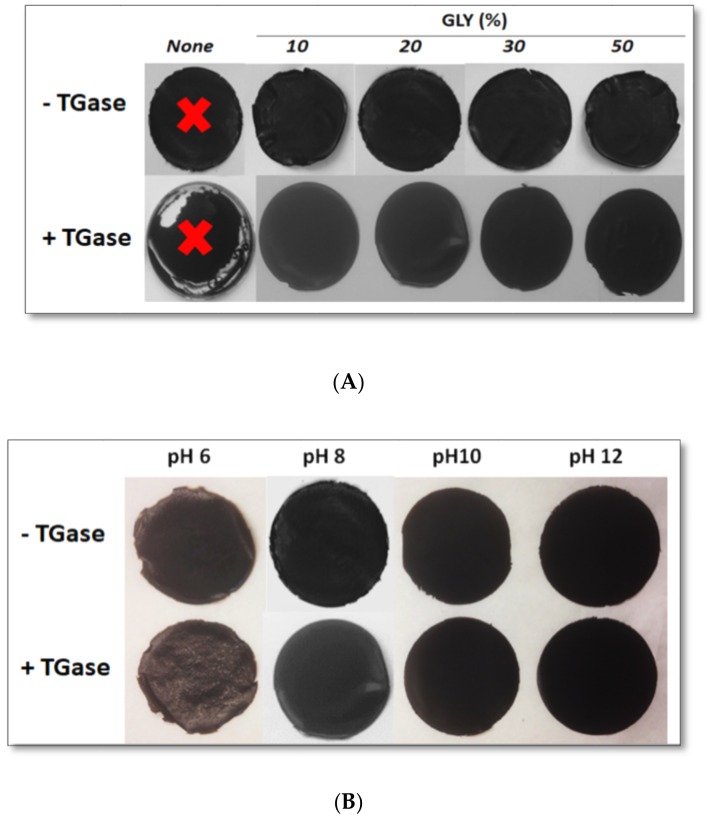
Images of protein concentrate (PC, 400 mg) films either containing different concentrations of glycerol (GLY) and obtained at pH 8.0 (**A**) or containing 20% GLY and obtained at different pH values (**B**) after incubation of the FFSs in the absence or presence of transglutaminase (TGase, 20 U/g protein). Further experimental details are given in the text.

PC FFSs either containing different GLY amounts or prepared at different pH values were incubated in the absence or presence of TGase and finally casted and dried for 48 h. Figure 4 shows that handleable black films were obtained at pH 8.0 by adding at least 10% GLY as plasticizer (panel A) and, in the presence of 20% GLY, at all the pHs between 6.0 and 12.0 (panel B).

**Figure 5:**
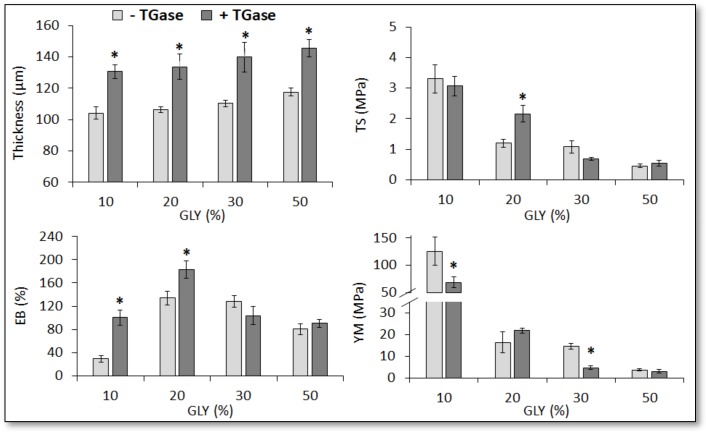
Effect of different concentrations of glycerol (GLY) on the mechanical properties (TS, tensile strength; EB, elongation at break; YM, Young’s modulus) of protein concentrate (PC, 400 mg) films obtained at pH 8.0 after incubation of FFSs in the absence or presence of transglutaminase (TGase, 20 U/g protein). Further experimental details are given in the text. * Significantly different values as compared to those obtained under the same experimental conditions in the absence of TGase (*p* < 0.05).

Figure 5 shows the effect of the different GLY concentrations on the mechanical properties of the films casted at pH 8.0, while Table 1 reports the same properties detected with films containing 20% GLY and casted at different pHs. Both experiments clearly indicate that the TGase-treated FFSs gave rise to significantly thicker films at all GLY concentrations and at all casting pH values. Moreover, the combined effects of pH and GLY concentrations resulted of particular interest. In fact, the addition of 20% GLY to FFS casted at pH 8.0 generated a peculiar positive effect on the mechanical properties of the films obtained with the TGase-treated FFS, causing a significant increase in their tensile strength (TS) and elongation at break (EB), without changing the quite low value of Young’s module (YM). 

**Figure 6:**
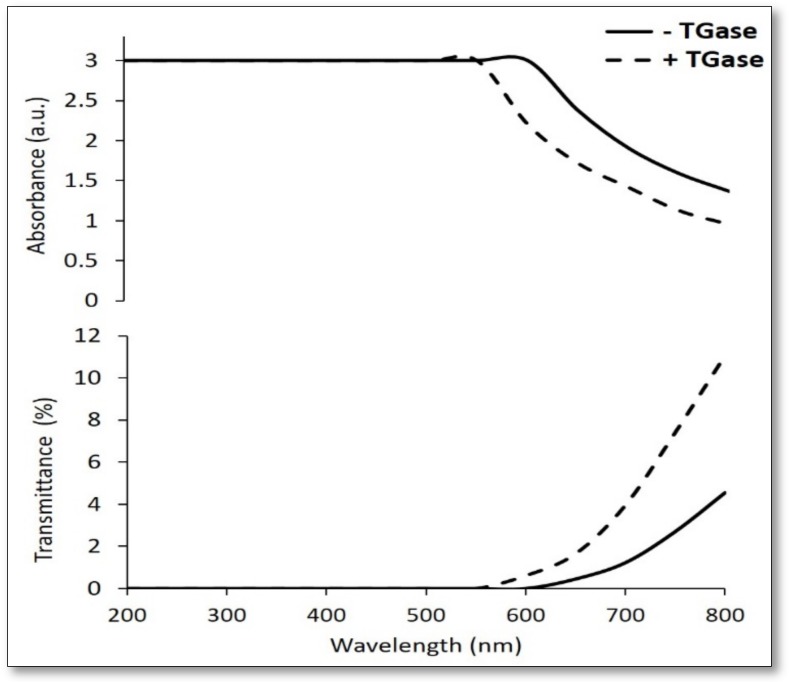
Absorbance and transmittance of films obtained from *Nigella sativa* defatted seed cake (*Ns*DSC) at pH 8.0 with 20% glycerol (GLY) after incubation of film forming solutions (FFSs) in the absence or presence of transglutaminase (TGase, 20 U/g protein). Further experimental details are given in the text.

The high absorbance and low transmittance of both kinds of film at 200–550 nm wavelengths (Figure 6) may be considered a further interesting feature of the *Ns*DSC-derived materials. In fact, they should be potentially able to hinder a possible photooxidative deterioration of the packaged foods and drugs by UV irradiation, as well as to suppress weeds and conserve water in crop production if used as biodegradable mulching sheets. Furthermore, a significant difference was also observed in the absorbance/transmittance intensity in the range of 550–800 nm of the films prepared from the FFS incubated in the presence of TGase with respect to those obtained from FFS incubated in the absence of enzyme. This result was probably due to the TGase-catalyzed change of the film protein network. 

Therefore, we can conclude that the enzymatically cross-linked *Ns*DSC-derived proteins may give rise at pH 8.0, in the presence of 20% GLY, to biodegradable, resistant, and at same time flexible dark films, exploitable not only for food packaging but also for mulching. In fact, it is well known that plastic mulch disposal is a distinguished environmental concern [32]. The black color of the obtained film would help not only to protect packaged drugs or food products from a variety of photooxidative agents, but also in the agriculture if used as mulching sheet. 

The permeability properties exhibited by the films obtained following FFS treatment with TGase confirmed the promising use of the *Ns*DSC-based material mostly for sheet mulching. In fact, the observed marked decrease in the barrier effect of the cross-linked films to both carbon dioxide and oxygen should allow a more active gas exchange without significantly influencing their poor water vapor (WV) permeability (Table 2). 

**Figure 7:**
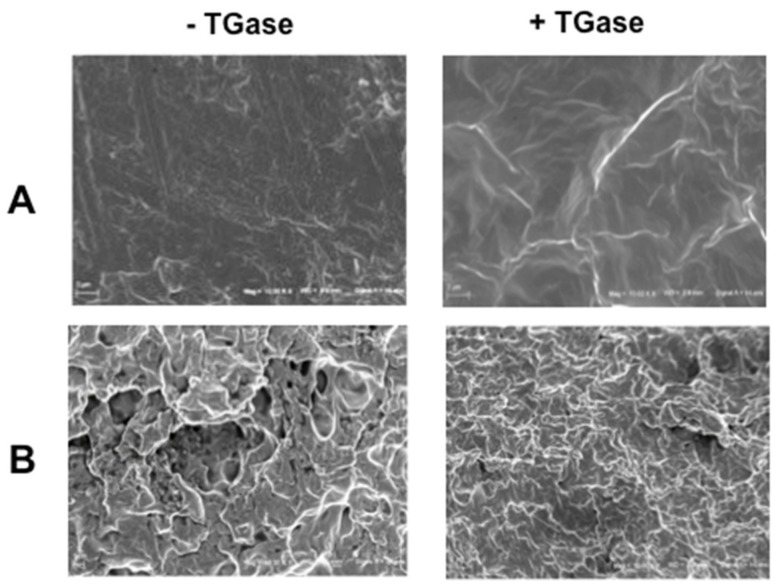
SEM analysis (10,000× magnification) of the surfaces (**A**) and cross-sections (**B**) of *Nigella sativa* defatted seed cake (*Ns*DSC)-derived films obtained in the absence (**A**) and presence (**B**) of transglutaminase (TGase)

Figure 7 shows the surface and cross-section morphology of the films containing 20% GLY and produced at pH 8.0 after FFS incubation in the absence or presence of TGase (20 U/g protein). Scanning electron microscopy (SEM) images indicate a smoother surface and a more continuous and homogeneous microstructure of the cross-section of the *Ns*DSC-based material obtained after FFS treatment with TGase, thus explaining the effect of the enzyme on the film mechanical and barrier properties.

It should be emphasized that the effects of the cross-links produced by TGase on the different properties of the protein-based films were extremely variable depending on the specific structural characteristics of the single proteins involved in the enzymatic reaction. In fact, as far as both mechanical and barrier properties are concerned, sometimes increases and other times decreases in the values of the individual measured parameters (TS, EB, and YM) were reported. The same variability was observed when permeabilities to gases or water vapor were analyzed in films containing different proteins able to act as TGase substrates [11,26,27,30,33,34,35,36,37,38].

**Figure 8:**
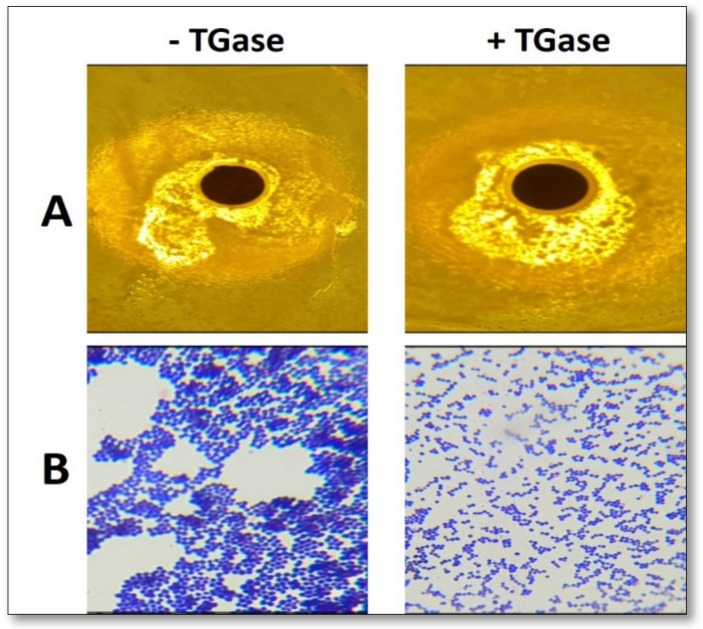
Antimicrobial properties of 20% glycerol (GLY)-containing protein concentrate (PC, 400 mg) films obtained from *Nigella sativa* defatted seed cake (*Ns*DSC) at pH 8.0 after incubation of film forming solutions (FFSs) in the absence or presence of transglutaminase (TGase, 20 U/g protein). The biological effect was evaluated on *Staphylococcus aureus* from American Type Culture Collection (ATCC) 25923 foodborne pathogenic bacterium by analyzing the growth of cells in direct contact with the film. Cells grown around PC films on mannitol salt agar after 18 h (**A**), Gram stain of the bacteria around the PC films (**B**). Further experimental details are given in the text.

Finally, all the *Ns*DSC-derived films exhibited, similarly to previously produced *Vicia ervilia* seed protein-based films [27,39], a clear antimicrobial activity tested towards *Staphylococcus aureus.* However, the inhibition zones produced by films prepared following FFS treatment with TGase were more regular and wider (Figure 8A), probably due to a more homogeneous diffusion of bioactive agents, such as polyphenols, tannins, and flavonoids, from the film cross-linked protein network. Moreover, a loopful of bacteria taken from the inhibition zones showed different arrangements, after Gram staining, when the two different kinds of film were analyzed (Figure 8B). Bacteria around films derived from FFSs treated with TGase were disintegrated and less condense with loose cells, probably as a consequence of a more homogeneous distribution into the cross-linked protein network of the bioactive agents possessing antimicrobial activity.

## 3. Materials and Methods

### 3.1. Materials 

*Ns*DSC was purchased from Alhathnawi General Trade Co. (Jenin, Palestine). GLY (about 87%) was supplied from the Merck Chemical Company (Darmstadt, Germany), whereas TGase (Activa WM), derived from the culture of *Streptoverticillium* sp., was supplied by Prodotti Gianni SpA (Milano, Italy). All other chemicals and solvents used in this study were analytical-grade commercial products. Bacterial strain *Staphylococcus aureus*, from American Type Culture Collection (ATCC 25923), was granted by Dr. M. Alqadi, An-Najah National University. BBL^TM^ mannitol salt agar (MSA) was purchased from Becton Dickinson and Company, France.

### 3.2. NsDSC Protein Extraction 

PC was obtained from *Ns*DSC by acid base extraction method as previously described [10] with some modifications. Dry *Ns*DSC was ground using a rotary mill (Grindomix GM200, Retsch GmbH, Haan, Germany) at a speed of 1200 rpm. for 5 min and the powder was dispersed in distilled water (1:10, *w*/*v*), brought to pH 12.0 with 1 N NaOH and stirred at medium speed for 2 h at room temperature. After centrifugation at 4000 rpm for 20 min, the supernatant was collected and the pH adjusted at 5.4 by 1 N HCl to form a precipitate which was then separated by centrifugation at 4000 rpm for 20 min. The pellet was then poured and uniformly distributed on a plastic plate and dried at 30 °C and 20% Relative Humidity (RH). The obtained PC was finally ground and the protein content (44%) was determined by the Kjeldahl’s method [40] using a nitrogen conversion factor of 6.25. 

### 3.3. Titration of PC from NsDSC

Z-average and zeta potential values of PC dissolved in distilled water (0.1 mg/mL) were determined by a Zetasizer Nano-ZSP (Malvern^®^, Worcestershire, UK) equipped with an automatic titrator unit (MPT-2). The device was equipped with a helium-neon laser of 4 mW output power operating at the fixed wavelength of 633 nm (wavelength of laser red emission). The instrument software programmer calculated the zeta potential through the electrophoretic mobility by applying a voltage of 200 mV using the Henry equation. The pH value of PC solution was adjusted at pH 12.0 by using 1.0 N NaOH and then the titration was carried out from pH 12.0 to pH 2.0 by adding 1.0, 0.5, and 0.1 N HCL as titrant solution under constant stirring at 25 °C. Z-average and zeta potential values were measured at each pH value in triplicate [10,41].

### 3.4. FFSs Preparation and Casting

To prepare the different FFSs, PC was dissolved in distilled water (4 g/100 mL) and the pH value was adjusted to pH 12.0 by using 1 N NaOH under constant stirring until the powder was completely solubilized. In preliminary experiments, PC solutions were added with different GLY amounts (10%–50% *v*/*w* protein) to find out the minimal GLY amount needed to obtain handleable films. Further experiments were carried out by adding 20% GLY (*v*/*w* protein) to aliquots of PC solution which were then incubated at 37 °C for 2 h at pH 8.0 in the absence or presence of microbial TGase (20 Units/g protein). All the FFSs (50 mL each containing 400 mg protein) were finally casted at different pH values (6, 8, 10, and 12) and, after 48 h, the dried films were analyzed.

### 3.5. Sodium Dodecyl Sulphate Polyacrylamide Gel Electrophoresis (SDS-PAGE)

The 250 µL of sample buffer (15 mM Tris–HCl, pH 6.8, containing 0.5% (*w*/*v*) SDS, 2.5% (*v*/*v*) GLY, 200 mM β-mercaptoethanol, and 0.003% (*w*/*v*) bromophenol blue), were added to aliquots of 0.1 mL of each FFS previously incubated at 37 °C for 2 h at pH 8.0 in the absence or presence of TGase (10 or 20 U/g protein), and analyzed by 12% SDS-PAGE. In all cases, SDS-PAGE was performed as previously described [42], at a constant voltage (80 V for 2–3 h). Coomassie Brilliant Blue R250 (Bio-Rad, Segrate, Milan, Italy) was used to stain the proteins and Bio-Rad Precision Protein Standards were simultaneously run as molecular weight markers.

### 3.6. Film Characteristics

*Ns*DSC films prepared with 20% GLY in the presence or absence of TGase were cut into 1-cm × 4-cm strips placed in a quartz cuvette and their light-barrier properties were determined by measuring their light absorption at wavelengths ranging from 200 nm to 800 nm, with a scan rate of 300 nm/min, using a Jenway UV-VIS spectrophotometer 6705 (Cole-Parmer Ltd., Staffordshire, UK). Film TS, EB, and YM values were determined in six specimens for each films (1 kN load and 5 mm/min speed), as previously reported by using an Instron universal testing instrument model No. 5543A (Instron Engineering Corp., Norwood, MA, USA) [43] after their conditioning at 25 °C and 50% RH for 2 h by placing the film samples into a desiccator over a saturated solution of Mg(NO_3_)_2_·6 H_2_O before being tested. Film thickness measurements were performed in six different points of each film with a digital micrometer (DC-516, sensitivity 0.1 μm). Film permeabilities to O_2_, CO_2_, and water vapor (WV) were detected in triplicate for each film, by using a Total Perm apparatus (ExtraSolution s.r.l., Pisa, Italy) endowed with barometric compensation, at 25 °C and 50% RH and at partial pressure differences of 1 bar for O_2_ and CO_2_ measurements and of 16 mbar for WV, respectively. In addition, aluminum masks were used with the aim to reduce the film test area to 5 cm^2^ [44,45,46]. *Ns*DSC film surface and cross-section ultrastructure was examined by SEM. Films were cut using scissors, mounted on stub, and sputter-coated with platinum-palladium (Denton Vacuum Desk V) before observation with Supra 40 ZEISS (EHT = 5.00 kV, detector in lens).

### 3.7. Film Antimicrobial Activity 

Bacterial strain of *Staphylococcus aureus* (ATCC 25923) was activated twice in a nutrient broth to reach a cell concentration corresponding to 0.5 turbidity at 600 nm, spectrophotometrically determined by measuring the amount of light scattered by the culture. Then, the cells were transferred to the selective media of mannitol salt agar to insure purity. A separated colony was retransferred to nutrient broth and incubated at 37 °C for 18 h to reach 0.5 ± 0.02 turbidity equivalent. Discs (5-mm diameter) obtained by cutting each film sample were used to evaluate the inhibition zones of *Staphylococcus aureus*. Then, 0.2 mL of the nutrient broth containing bacterial cells was loaded on the mannitol salt agar Petri dishes (five discs of each sample were distributed over the Petri dish) and, then, all dishes were incubated at 37 °C for 18 h. Finally, the inhibition zones were observed and evaluated.

### 3.8. Statistical Analysis 

JMP software 10.0 (SAS Institute, Cary, NC, USA), one way ANOVA, and the least significant difference test for mean comparisons were used. Differences were considered to be significant at *p* < 0.05. 

## 4. Conclusions

The development of new bio-materials from agro-industrial wastes is one of the major technological challenges, and the defatted seed cakes, by-products of oil producing industry, may be good and cheap sources of both energy and protein. The present study focused on the possibility to obtain potentially useful protein-based biodegradable/edible films by recovery and formulation procedures from seed oil cakes, offering a sustainable and eco-friendly option to produce novel prototypes for applications others than animal feed, fertilizers, or culinary food. Thus, innovative and biodegradable/edible black films from *Nigella sativa* defatted seed cakes were obtained. The GLY-plasticized films containing enzymatically cross-linked proteins showed mechanical and barrier properties, as well as antimicrobial ones, compatible with a possible re-use of the *Nigella sativa* defatted seed cakes as a renewable source to produce bioplastics of food and agricultural interest. Potential applications of the obtained bio-materials (e.g., coating/wrapping of different fabricated foods for shelf-life extension, protection of fruits and vegetables by control of maturation, soil mulching for a more sustainable agricultural production) deserve to be investigated.

## Figures and Tables

**Table 1:** Mechanical properties (TS, tensile strength; EB, elongation at break; YM, Young’s modulus) of 20% glycerol (GLY)-containing protein concentrate (PC, 400 mg) films obtained at different pH values after film forming solution (FFS) incubation in the absence or presence of transglutaminase (TGase, 20 U/g protein). Further experimental details are given in the text.

Film	pH	Thickness (µm)	TS (MPa)	EB (%)	YM (MPa)
**− TGase**	6.0	106.5 ± 3.9	1.2 ± 0.2	9.8 ± 2.7	94.4 ± 5.0
	8.0	107.5 ± 2.8	1.4 ± 0.2	130.4 ± 8.4	19.4 ± 2.8
	10.0	105.2 ± 3.8	1.3 ± 0.3	40.9 ± 8.5	45.4 ± 3.5
	12.0	109.2 ± 7.2	1.8 ± 0.2	35.2 ± 2.3	55.5 ± 7.8
**+ TGase**	6.0	135.0 ± 8.4 *	1.8 ± 0.3	16.4 ± 3.2 *	85.7 ± 5.3
	8.0	134.9 ± 8.4 *	2.2 ± 0.1 *	183.5 ± 8.5 *	18.7 ± 2.3
	10.0	139.8 ± 7.8 *	1.8 ± 0.2	70.8 ± 7.2 *	38.9 ± 4.6
	12.0	131.6 ± 7.3 *	2.3 ± 0.4	61.0 ± 3.8 *	30.0 ± 4.5 *

* Significantly different values as compared to the ones obtained under the same experimental conditions in the absence of TGase (*p* < 0.05).

**Table 2:** Gas and water vapor (WV) barrier properties of 20% glycerol (GLY)-containing protein concentrate (PC, 400 mg) films obtained at pH 8.0 after film forming solutions (FFS) incubation in the absence or presence of transglutaminase (TGase, 20 U/g protein). Further experimental details are given in the text.

Film	CO_2_	O_2_	WV
	(cm³ mm m^−^² day^−^¹ kPa^−1^)		
**− TGase**	1.1 ± 0.2	0.04 ± 0.01	0.004 ± 0.001
**+ TGase**	4.6 ± 0.7 *	4.16 ± 0.13 *	0.002 ± 0.001

* Significantly different values as compared to those obtained under the same experimental conditions in the absence of TGase (*p* < 0.05).
